# Host range of the oothecal parasitoid *Aprostocetus hagenowii* (Hymenoptera: Eulophidae)

**DOI:** 10.1093/jisesa/ieaf001

**Published:** 2025-01-23

**Authors:** Chelsea M Smith, Henry Y Fadamiro, Arthur G Appel

**Affiliations:** Department of Biological and Environmental Sciences, Troy University, Troy, AL, USA; Department of Entomology and Plant Pathology, Auburn University, Auburn, AL, USA; Department of Entomology, Texas A&M University, College Station, TX, USA; Department of Entomology and Plant Pathology, Auburn University, Auburn, AL, USA

**Keywords:** parasitoids, Hymenoptera, Blattodea, urban pests, host-parasitoid interaction

## Abstract

*Aprostocetus hagenowii* (Ratzburg) is a generalist parasitoid of cockroach (Blattodea) oothecae. Previous studies examining the host range of *A. hagenowii* have largely focused on cockroaches of economic and medical importance, which represent a minority of species in an order filled with species of diverse morphology, behavior, and ecology. The aim of this study was to expand the known host range of *A. hagenowii* with emphasis on nonpest as well as pest species from 3 cockroach families (Blattidae, Corydiidae, and Ectobiidae). Previously recorded host species were also reexamined. Oothecae from 17 cockroach species were exposed to *A. hagenowii*. Three new host species were recorded: *Blatta lateralis* (Walker) (Blattidae), *Neostylopyga propinqua* (Shelford) (Blattidae), and *Parcoblatta fulvescens* (Saussure and Zehntner) (Ectobiidae). Among the reexamined host species *Periplaneta australasiae* (Fab.) (Blattidae), *Blatta orientalis* L. (Blattidae), and *Neostylopyga rhombifolia* (Stoll) (Blattidae) were successfully parasitized. The cuticle thicknesses of 7 cockroach species’ oothecae were also investigated. There were significant differences [Kruskal–Wallis: each zone (below keel, side, and bottom) measured *P* < 0.001] in cuticle thickness among the species measured. *Polyphaga sassurei* (Dohrn) (Corydiidae) and *Eurycotis floridana* (Walker) (Blattidae) had the thickest cuticles (each zone >0.09 mm) and *Blattella germanica* (L.) (Ectobiidae) had the thinnest (each zone <0.03 mm). However, the mean *A. hagenowii* ovipositor length (0.92 mm ± 0.01 mm) far exceeded the thickest oothecae measured. Oothecal cuticle thickness alone was not observed to determine the host suitability of each tested cockroach species for *A. hagenowii*.

## Introduction

*Aprostocetus hagenowii* (Ratzeburg) is a generalist parasitoid wasp that parasitizes the oothecae of cockroaches (Blattodea) (LeBeck 1991). Most known hosts are peridomestic pest species in the family Blattidae, such as the American cockroach *Periplaneta americana* (L.), the Oriental cockroach *Blatta orientalis* L., the harlequin cockroach *Neostylopyga rhombifolia* (Stoll), and the Florida woods cockroach *Eurycotis floridana* (Walker) (LeBeck 1991). An unidentified species of wood cockroach in the genus *Parcoblatta* (Ectobiidae) was also reported as a host for *A. hagenowii* (Edmunds 1952). The German cockroach *Blattella germanica* (L.) (Ectobiidae) has been listed as a host as well, but the consensus is that these reports are erroneous (Roth and Willis 1960, LeBeck 1991).

Investigations of parasitoid–host ranges are often conducted in relation to species of importance from the human perspective—to find new pest host species and to determine what nontarget organisms would be at risk when the parasitoid is released for pest control (Van Driesche 2004). Most host range studies for *A. hagenowii* have followed this pattern (Roth and Willis 1954, 1960, Narasimham and Sankaran 1979, Harlan and Kramer 1980, Suiter et al. 1996). Pest cockroaches represent less than 1% of total cockroach species diversity (Roth and Willis 1960, Bell et al. 2007). Thus, past studies focused on this group have almost certainly failed to provide a greater understanding of the true host range of *A. hagenowii* or related aspects, such as coevolution and ecology.

The inclusion of nonpest insect species in parasitoid–host range studies has several obstacles. Funding focused on the ecology, biology, and natural history of nonpest insect species is limited (Slade and Ong 2023). Exotic nonpest species may be harder to obtain, especially if they are native to a country or region outside of the researcher’s own, and importation of exotic organisms often requires a permitting process through governmental entities, such as the United States Department of Agriculture (USDA): Animal and Plant Health Inspection Service (https://efile.aphis.usda.gov/s/). Furthermore, these species may lack established rearing techniques or may be adapted to environments that are difficult to replicate in a laboratory setting.

Over the past 2 decades, the proliferation of hobbyists involved in the online discussion and trade of exotic arthropods has made it easier for researchers to obtain nonpest species and instruction on their rearing. Access to additional exotic and nonpest cockroach species would make for a more thorough investigation of the host range and coevolutionary relationship between *A. hagenowii* and cockroaches. Testing cockroach species that share their native ranges with *A. hagenowii* should be a priority. However, the exact native range of *A. hagenowii* is unknown because it currently has a cosmopolitan distribution, and it may have originated from sub-Saharan Africa alongside many of its known hosts in the genus *Periplaneta* (Bell and Adiyodi 1981).

The objective of this study was to provide a more thorough examination of the host range for *A. hagenowii*. Cockroach species from 3 families (Blattidae, Ectobiidae, and Corydidae) with diverse geographic origins, natural histories, and oothecal morphologies were selected for testing. Under natural conditions, cockroach oviposition behaviors, such as burial and guarding, may prevent *A. hagenowii* from accessing potential hosts. Considering that our colonies are raised in artificial environments, and there is variation in how individual cockroaches oviposit (McKittrick 1964, Smith et al. 2022), we provided unconcealed oothecae to *A. hagenowii*. The inclusion of nonpest species, alongside pest species and previously recorded hosts, was a focus of this study.

## Methods and Materials

### Wasps

*Aprostocetus hagenowii* wasps were provided by Dr. Barry Pawson (PNE, Inc., Tipp City, OH; Retired). This strain of *A. hagenowii* was originally brought into the culture in 1993 using individuals collected in Texas (Pawson and Gold 1993). The colony used in this study has been reared at Auburn University since 2020. Our colony was maintained in 100 mm × 15 mm polystyrene Petri dishes on the oothecae of *P. americana*. *Aprostocetus hagenowii* is an arrhenotokous species (unmated females produce only male offspring) (LeBeck 1991), and a postemergence mating period of 24 h was used to allow for adequate mating time to occur. Individual female wasps, which are much larger than males and possess short antennal setae, were identified, separated into Petri dishes, and each provided with 1 to 3 fresh oothecae (aged 1 to 3 wk). The dishes were then placed into a growth chamber [25 ± 2 °C, 30% to 40% RH, and a 16:8 (L:D) photoperiod] for incubation, which takes about 1 mo. Adult wasps were not provided with food or water because the nutrients required for parasitization are sequestered during larval development (personal communications with Dr. Pawson).

### Cockroaches

The cockroach species used throughout the study came from numerous sources including online retailers, private collectors, and laboratory colonies. The origin and husbandry history of species from retailers and collectors is unclear and typically not provided on store pages. Most of the pest cockroach species from Auburn University have been reared there since 1985 and were obtained from colonies at University of California Riverside. The scientific and common names, authority, family, origin, year obtained, and reported host status for *A. hagenowii* of each cockroach species are provided in Table 1. All cockroaches were provided a diet of Purina Laboratory Diet 5001 rat chow blocks, Purina Dog Chow (Ralston Purina, St. Louis, MO), and water ad libitum. Raw carrot, romaine lettuce, and Wardley Goldfish Flake Food (Hartz Mountain Corp., Secaucus, NJ, USA) were provided ad libitum to supplement their diet. The following species were reared in 7.6 l (2.0 gal) plastic buckets to make oothecal collection easier: *P. americana*, *Blatta lateralis* (Walker), and the brownbanded cockroach, *Supella longipalpa* (Fab.)*. Perplaneta americana* and *B. lateralis* were provided with loosely rolled 0.64 cm (0.25 in) 23-gauge hardware cloth for harborage. The bottom of the bucket which housed *P. americana* was removed and replaced with a layer of hardware cloth. Oothecae that fell through the openings were collected in a second bucket underneath. The buckets holding colonies of *S. longipalpa* contained hanging strips of canvas for harborage. The strips were suspended from a wire running through the center of rubber flask stoppers bolted to the upper bucket wall. *Arenivaga bolliana* (Sassure), *Polyphaga aegyptiaca* (L.), and *Po. sausseri* (Dohrn) are relatively large burrowing species. They were reared in L × W × H: 35.6 cm × 22.9 cm  × 12.7 cm (14.0 in. × 9.0 in. × 5.0 in.) plastic shoe boxes that were half-filled with ground coconut fiber. The tops of both boxes were covered by a screen held in place with rubber bands. *Parcoblatta lata* (Brunner von Wattenwyl) and *Pa. fulvescens* (Sassure and Zehntner) were reared in similar boxes that measured L × W × H: 34.3 cm  × 20.3 cm  × 12.7 cm (13.5 in.  × 8.0 in.  × 5.0 in.), with expanded polystyrene (EPS; Styrofoam) and cardboard sheets as shelter and ground coconut fiber as an oviposition substrate. A solid plastic lid was used to maintain the higher humidity preferred by these species.

**Table 1.** A list of the cockroach species, number of oothecae (*n*) from each exposed to *A. hagenowii*, host status, and parasitism success during host range testing

Species and authority	*n*	Common name	Family	Origin, year obtained	Host status	Parasitism success %
*Blatta lateralis* (Walker)	8	Turkestan Cockroach	Blattidae	AU, 1990	N	62.5%
*Blatta orientalis* L.	9	Oriental Cockroach	Blattidae	PC, 2021	P, R	77.8%
*Deropeltis paulinoi* Bolívar	6	Ornate Velvet Cockroach*	Blattidae	OR, 2021	…	
*Neostylopyga* *propinqua* (Shelford)	25	African Bullet Cockroach*	Blattidae	PC, 2020	N	16.0%
*Neostylopyga* *rhombifolia* (Stoll)	6	Harlequin Cockroach	Blattidae	PC, 2022	P, R	33.3%
*Periplaneta australasiae* (Fab.)	3	Australian Cockroach	Blattidae	AU, 1985	P, R	66.7%
*Periplaneta brunnea* Burmeister	3	Brown Cockroach	Blattidae	AU, 1985	P	
*Periplaneta fuliginosa* (Serville)	3	Smokybrown Cockroach	Blattidae	AU, 1985	P	
*Periplaneta japonica* Karny	3	Japanese Cockroach*	Blattidae	PC, 2022	P	
*Eurycotis floridana* (Walker)	4	Florida Woods Cockroach*	Blattidae	OR, 2021	P	
*Eurycotis lixa* Rehn	4	Hustler Cockroach*	Blattidae	OR, 2021	…	
*Arenivaga bolliana* (Saussure)	7	Boll’s sand cockroach*	Corydiidae	AU, 2015	…	
*Polyphaga aegyptiaca* (L.)	5	Egyptian Desert Cockroach*	Corydiidae	OR, 2021	…	
*Blattella germanica* (L.)	5	German Cockroach	Ectobiidae	AU, 1985	…	
*Parcoblatta fulvescens* (Saussure and Zehntner)	6	Fulvous Wood Cockroach*	Ectobiidae	PC, 2020	N	33.3%
*Parcoblatta lata* (Brunner von Wattenwyl)	16	Broad Wood Cockroach*	Ectobiidae	PC, 2020	…	
*Supella longipalpa* (Fab.)	17	Brownbanded Cockroach	Ectobiidae	AU, 1993	…	

Species with common names marked by an asterisk have not been assigned official common names by the Entomological Society of America as of June 2023. Host Status: P = Previously recorded as host, R = Reconfirmed as host, - = not previously recorded as host, and N = newly recorded host. Origin key: AU = Auburn University, OR = online retailer, PC = private collector.

The following species were reared in 1.9 l (0.5 gal) glass jars containing approx. 2.5 cm (1 in) of ground coconut fiber: *N. rhombifolia*, *N. propinqua* (Shelford), and *Deropeltis paulinoi* Bolivar. The remaining species were housed in 3.8 l (1 gal) glass jars: *Bl. germanica*, *Periplaneta japonica* Karny, *P. australasiae* (Fab.), *P. fuliginosa* (Serville), *P. brunnea* Burmeister, *B. orientalis*, *E. floridana*, and *E. lixa* Rehn. The species reared in glass jars were provided with corrugated cardboard harborage (flat sheets or rolls). Jars housing species preferring higher humidity were misted with water twice per week and provided with an additional substrate such as leaflitter and extra ground coconut fiber 2.5 cm to 6 cm (1 in. to 2 in.) deep. A band of mineral oil applied to the inner, upper surface of each jar prevented escape. The jar openings were covered with a layer of cloth mesh and a paper towel both held in place with rubber bands. The oothecae of *N. rhombifolia*, *Pa. lata*, and *Pa. fulvescens* were especially prone to desiccation upon removal from their respective colony containers. Desiccation was prevented by placing the oothecae on moistened Kimwipes (Kimberly-Clark, Neenah, WI, USA) while in Petri dishes. All cockroach colonies and cockroach oothecae collected prior to exposure to *A. hagenowii* were held in a room maintained at 26 ± 2 °C, 40% to 50% RH, and a 16:8 (L:D) photoperiod.

### No-Choice Assays

The timing of wasp emergence and oviposition of oothecae heavily influenced the availability of each for testing. The no-choice assays entailed pairing 1 to 10 female *A. hagenowii* (aged at least 24 h postemergence) with individual oothecae in separate Petri dishes. While individual female *A. hagenowii* often readily parasitize *P. americana*, we observed that females in a group were more likely to interact with potential host oothecae from other species (personal observations). *A. hagenowii* appears to lack a host age preference, but parasitism success declines as host age increases (Hagenbuch et al. 1988, Tee and Lee 2017). Thus, the oothecae selected for use in the assays were aged ≤14 d, with the exception of *A. bolliana* oothecae, which were obtained from an unmonitored colony under the care of the Appel lab. Oothecae were not previously exposed to oothecal parasitoids. Oothecae free of defects (ie, broken or missing keel, dimples, or other visible abnormalities, etc.) were selected for experiments. However, the oothecae collected from the *P. japonica, P. brunnea, E. floridana, E. lixa*, and *N. rhombifolia* colonies were often malformed and dimpled limiting their use in the no-choice experiments. In these cases, oothecae with the fewest defects were selected for exposure to *A. hagenowii*. *Bl. germanica* oothecae are especially prone to desiccation when prematurely separated from the mother’s abdomen, which would likely cause the cockroach nymphs and parasitoid larvae to die before completing development. However, the presence of the mother may also deter *A. hagenowii* from interacting with attached ootheca. Oothecae of *Bl. germanica* provided to *A. hagenowii* in either condition were not expected to be successfully parasitized, but data on parasitoid–host interactions could still be gathered.

Each assay was observed for 30 min after provisioning of an ootheca for signs of host investigation and acceptance (eg, antennal drumming, ovipositor tapping, and attempted oviposition). The Petri dishes were then transferred to a stinging cage in the incubator and were checked every other hour during the first day of exposure for wasps displaying oviposition behavior. Upon the death of the wasps, the oothecae in the dishes were monitored for the emergence of wasps and nymphs. Dishes that produced wasps were marked with the emergence date, and dishes that produced cockroach nymphs were discarded. Dishes that produced neither wasps nor nymphs after 2 mo of incubation were dissected to determine if *A. hagenowii* oviposition may have occurred.

*A. hagenowii* searches for and interacts with hosts more readily if many wasps are present simultaneously (personal observations). Species that initially garnered little interest from *A. hagenowii* or which failed to produce wasps after oviposition behaviors were observed were selected for additional testing in *A. hagenowii* colony cages. These additional no-choice tests were performed by exposing multiple oothecae of the same cockroach species to *A. hagenowii* by placing them in an open Petri dish inside a Plexiglas stinging cage that housed numerous (≥60) *A. hagenowii* wasps of mixed sex and age. Hosts that are superparasitized (parasitized by multiple parasitoids of the same species) by *A. hagenowii* tend to produce large numbers of offspring, a less female-biased sex ratio, and smaller individual wasps, but they are typically able to complete development (Hagenbuch et al. 1988, personal observations). In the unlikely event that an ootheca was too heavily parasitized to support the development and emergence of adult *A. hagenowii*, dissection of the oothecae would have revealed the presence of larvae. The stinging cage was held in the wasp growth chamber throughout each assay. The oothecae were observed for at least 30 min for signs (described above) of oviposition interest from the wasps. Periodic monitoring of the wasps for oviposition behavior was carried out until all the wasps in the cage had died. The Petri dishes were then closed and incubated within the wasp growth chamber. Oothecae that produced wasps, nymphs, or neither were recorded, discarded, or dissected as described above.

### Ootheca Cuticle Characteristics and Ovipositor Length

The oothecae produced by 7 cockroach species were selected based on availability and represented host and nonhost species from each of the 3 families under investigation. The majority of the species selected (*Bl. germanica, Pa. fulvescens, N. rhombifolia, B. lateralis*, and *E. floridana*) were also used in the no-choice host range experiment. However, the oothecae of *P. americana* and *Po. saussurei* (Dohrn) were also used. *Periplaneta americana* was selected because it is the host used for maintaining the *A. hagenowii* colony, and *Po. saussurei* was used in place of its congener *P. aegyptiaca* due to the latter’s colony having too few individuals. The cuticle thickness of each species’ oothecae was measured with an advanced onsite sensor Absolute Digimatic electric caliper (Mitutoyo America Corp., Aurora, IL, USA). The caliper was calibrated with precision stainless steel ring shims (McMaster-Carr, Elmhurst, IL, USA), with the smallest tested shim being 0.1 mm thick. The manufacturer lists a tolerance of ±0.013 for the 0.1 mm shim. The caliper measurements of the 0.1 mm shim were conducted in the same manner used to measure cuticle thickness (described below) and returned a mean thickness of 0.083 mm (standard error of the mean (SEM) ± 0.002, *N* = 4); thus, 0.016 mm was added to measurements of oothecal cuticle thickness to compensate.

**Fig. 1.**
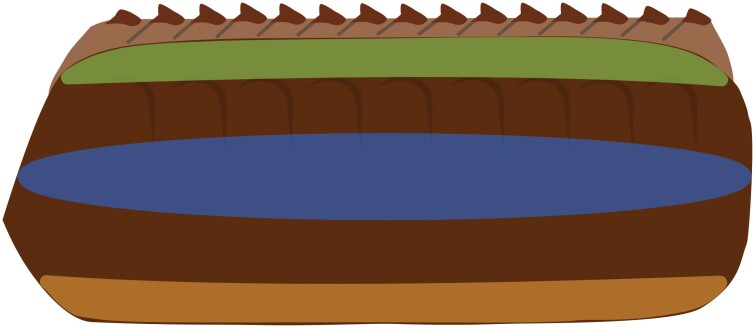
Diagram of areas measured to determine thickness of the oothecal cuticle: green—below keel, blue—side, and orange—bottom.

Oothecae were cut in half along and through the keel with a 5 mm micro knife (Fine Science Tools, North Vancouver, B.C.). Fine-tipped forceps were used to remove the oothecal contents from each half and to position the cuticle in the caliper jaws. Once in place, the caliper jaws were gently closed and placed within a 3-prong clamp. A support was placed beneath the caliper’s main scale to hold it level with the clamp. The clamp screws were fully tightened around the caliper jaws, then loosened until pressure was no longer applied. The resulting measurement was recorded and the process was repeated 2 additional times with the same cuticle piece to obtain an average thickness. *A. hagenowii* has been observed ovipositing on the sides and bottoms of host oothecae, but it is unknown if the cuticle thickness of an ootheca is consistent throughout its structure. The areas below the keel, at the side, and bottom (Fig. 1) of each ootheca were measured to determine if cuticle thickness varied by location.

An ocular micrometer mounted to a dissecting microscope was calibrated with a stage micrometer and used to measure *A. hagenowii* ovipositor length. The ovipositor of *A. hagenowii* is held in a groove that runs the length of the abdomen and is shaped similarly to an insect or sewing pin. To isolate the ovipositor, the abdomen of a dead female *A. hagenowii* wasp was gently crushed, and the ovipositor was held in place with fine-tipped forceps while the abdominal debris was swept away with a paintbrush. The length of each ovipositor was determined by measuring from the pointed tip of the distal end to just under the bulb (swelling) at the proximal end.

### Statistical Analysis

Cuticle thickness measurements were analyzed with a Kruskal–Wallis test and Dunn’s post hoc test in JASP (Version 0.16.3; JASP Team 2022) (Goss-Sampson 2022). Parasitism success was calculated as the proportion of exposed oothecae that produced wasps. The ANOVA and post hoc test results were compared to a threshold of *α* = 0.05 and, with the exception of parasitism success, data are described as means ± SEM.

## Results

### Host Range

*A. hagenowii* successfully parasitized 3 newly recorded host species: *B. lateralis*, *N. propinqua*, and *Pa. fulvescens*. Of the 8 *B. lateralis* oothecae provided, 62.5% were successfully parasitized, and the average development time (time to emergence) for wasps within *B. lateralis* oothecae was 32.4 d. *A. hagenowii* had a 16.0% parasitism success rate on *N. propinqua* oothecae and an average development period of 38.3 d. Lastly, parasitism success when provided *Pa. fulvescens* oothecae was 33.33%, and development time on this host species averaged 42.5 d.

Among the previously recorded hosts that were reexamined, only *N. rhombifolia*, *P. australasiae*, and *B. orientalis* were successfully parasitized. Parasitization of *N. rhombifolia* (33.33%) only occurred when the oothecae were provided to *A. hagenowii* in the stinging cage. *Polyphaga aegyptiaca* and *P. japonica* oothecae were also provided in the stinging cage but were not successfully parasitized. Due to a mismatch in wasp and oothecae availability, the number of *P. australasiae* oothecae available for testing was very limited, but the majority (66.67%) exposed produced wasps. *Aprostocetus hagenowii* was most successful at parasitizing *B. orientalis* (77.78%). *Periplaneta brunnea*, *P. fuliginosa*, *P. japonica*, and *E. floridana* produced few oothecae that were free of defects (eg, damaged keels, large dimples, etc.), limiting the number of replicates that could be provided to *A. hagenowii*. *Eurycotis lixa* and *Po. aegyptiaca*, had low replicate numbers due to low oothecal production, and *Bl. germanica* underwent limited testing due to challenges in keeping oothecae from desiccating after detachment from their mothers. Dissections of exposed oothecae that failed to produce wasps or cockroach nymphs after 2 months of incubation showed no signs of attempted parasitism.

*Aprostocetus hagenowii* displayed signs of interest (antennal drumming, ovipositor tapping) toward 15 of the 17 species of cockroach provided. Furthermore, *A. hagenowii* displayed interest in oothecae despite the presence of defects. The oothecae of *S. longipalpa* and *Bl. germanica* (2 attached to mother and 3 detached) were exceptions as *A. hagenowii* would only make brief contact with their oothecae or ignore them completely. The replicate number of oothecae from each cockroach species is provided in Table 1.

### Oothecal Cuticle Thickness and Ovipositor Length

The oothecae from the 7 species investigated had significantly different cuticle thicknesses for each zone measured [Kruskal–Wallis: below keel (*H*(5) = 21.762, *P* < 0.001), side (*H*(6) = 34.111, *P* < 0.001), bottom (*H*(6) = 34.299, *P* < 0.001)] (Fig. 1). Replicate number and mean thickness of each species’ oothecae is provided in Table 2. *Po. saussurei* and *E. floridana* had the thickest cuticles (all zones >0.09 mm). Along with *P. americana*, they formed a ‘thick cuticle’ group with all having mean measurements >0.08 mm (Table 2). *Neostylopyga propinqua*, *B. lateralis*, *Pa. fulvescens*, and *Bl. germanica* oothecae formed the second ‘thin cuticle’ group with mean cuticle thicknesses for each zone <0.07 mm (Table 2). The zone below the keel of *Bl. germanica* oothecae was excluded from the results because it was too narrowly separated from the side of the ootheca for measurement with the calipers. *Bl. germanica* had the thinnest cuticle overall with the side and bottom both <0.03 mm in thickness. The mean *A. hagenowii* ovipositor length was 0.92 ± 0.012 mm (*N* = 6).

**Table 2.** Mean oothecal cuticle thickness measurements of several cockroach species

Species (*n*^oothecae^)	Below keel (mm ± SEM)	Side (mm ± SEM)	Bottom (mm ± SEM)
*Blatta lateralis* (6)	0.065 ± 0.005	0.057 ± 0.004	0.052 ± 0.004
*Blattella germanica* (6)	N/A	0.027 ± 0.001	0.027 ± 0.002
*Eurycotis floridana* (4)	0.093 ± 0.007	0.110 ± 0.007	0.110 ± 0.006
*Neostylopyga rhombifolia* (5)	0.059 ± 0.007	0.050 ± 0.004	0.058 ± 0.003
*Parcoblatta fulvescens* (6)	0.047 ± 0.011	0.043 ± 0.005	0.050 ± 0.008
*Periplaneta americana* (7)	0.105 ± 0.007	0.087 ± 0.010	0.081 ± 0.008
*Polyphaga sausserei* (8)	0.098 ± 0.008	0.112 ± 0.004	0.120 ± 0.005

## Discussion

Oothecae from 17 species of cockroach were exposed to *A. hagenowii*. Three of these species (*B. lateralis*, *N. propinqua*, and *Pa. fulvescens*) are new host records, and 3 previously recorded host species (*B. orientalis*, *N. rhombifolia*, and *P. australiasiae*) are reconfirmed as hosts (LeBeck 1991). *Bl. lateralis* and *N. propinqua* are unsurprising additions to the host range for *A. hagenowii*. Both are members of Blattidae and have congeners (*B. orientalis* and *N. rhombifolia*) that were previously recorded as hosts (Pemberton 1941, Usman 1949). *Parcoblatta fulvescens* is an unusual host for *A. hagenowii*. It is a member of the Ectobiidae, endemic to North America (Atkinson et al. 1991), and rarely deposits its oothecae in locations accessible to *A. hagenowii* (Horn and Hanula 2002, Personal observations). *Aprostocetus hagenowii* appears able to locate oothecae buried under lightweight particles, such as coconut fiber, sand, or expanded polystyrene foam but is unable to move those materials (Smith et al. 2022; personal observations). *Parcoblatta fulvescens* as well as other *Parcoblatta* sp. can oviposit in substrates (eg, soil, rotting logs, etc.) (Horn and Hanula 2002) that are likely to act as barriers to *A. hagenowii*. A non-native biological control organism parasitizing a native nontarget species is concerning, but it is unlikely that *A. hagenowii* will have a meaningful impact on *Pa. fulvescens* populations owing to the latter’s ootheca concealing behavior and the former’s low parasitism rate when provided unconcealed *Pa. fulvescens* oothecae.

The initial parasitism success of *A. hagenowii* on *B. lateralis* oothecae led to the creation of an additional study (Smith et al. 2023) investigating the potential of *A. hagenowii* as a biological control for this cockroach, which is a pest of increasing concern throughout the US Southwest (Gaire and Romero 2020). Smith et al. (2023) was able to rear *A. hagenowii* for multiple generations using only *B. lateralis* oothecae for hosts. Similar attempts during the current study to rear *A. hagenowii* on *N. propinqua* oothecae for multiple generations failed to produce wasps beyond a second generation. Key differences between the oothecae of the 2 species are that *N. propinqua* oothecae are about half the size of *B. lateralis* oothecae and more prone to desiccation (personal observations), which may limit the viability of *N. propinqua* oothecae for *A. hagenowii* larval development.

There are many possible explanations for why *A. hagenowii* failed to parasitize 11 of the 17 species of cockroach oothecae provided. Oothecal quantity and quality varied widely among the species tested. Low replicate number (≤5 oothecae) is one possible explanation for why parasitism was not observed in *P. brunnea*, *P. fuliginosa*, *P. japonica*, and *E. floridana* despite their being previously recorded hosts (LeBeck 1991, Dai 1992). The *A. hagenowii* strain used in this study has been reared on *P. americana* oothecae for over 30 years, which may have affected its preferences and ability to switch to other host species (Smith et al. 2023). The characteristics of the oothecal cuticle, the environment within the ootheca, oothecal age, and the composition of each species’ eggs likely also play roles in host acceptance and parasitism success. Vinson (2010) notes that the composition, amount, and quality of the nutrition held within a host’s egg plays a major role in its ability to support a developing parasitoid larva. Egg quality also changes as the host embryo develops—proteins, tissues, and structures become more complex—which may affect the development and success of the parasitoid (Vinson 2010). While *A. hagenowii* appears to lack host age preferences for *P. americana* oothecae, parasitism success sharply declines as hosts near hatching (Tee and Lee 2017).

**Fig. 2.**
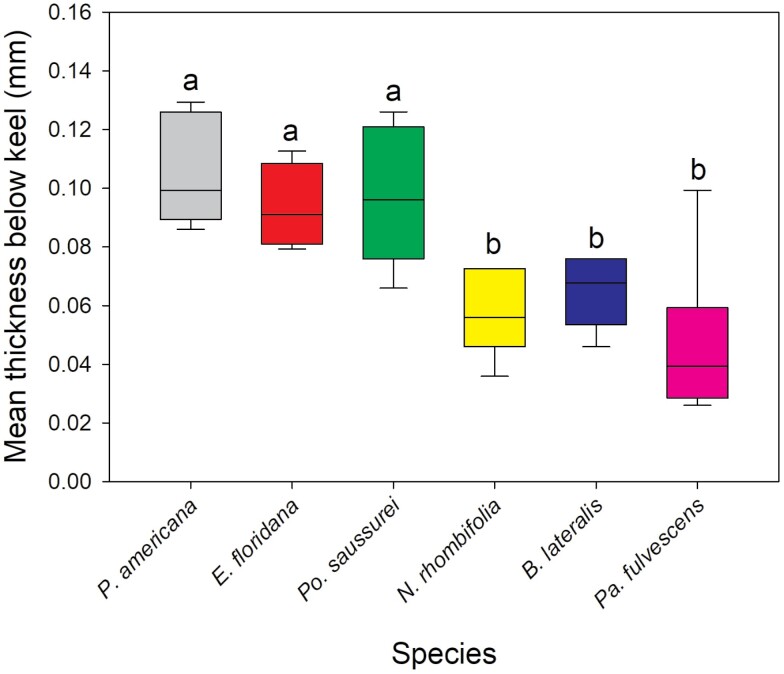
Kruskal–Wallis test and Dunn’s post hoc comparison of mean oothecal cuticle thickness measurements below the keel. The below keel zone of *Bl. germanica* is excluded as it was too narrow to measure. Boxes that do not share letters are significantly different with *P* ≤ 0.05. Number of oothecae measured (replicate): *P. americana* = 6, *E. floridana* = 4, *Po. saussurei* = 8, *N. rhombifolia* = 5, *B. lateralis* = 6, and *Pa. fulvescens* = 6.

**Fig. 3.**
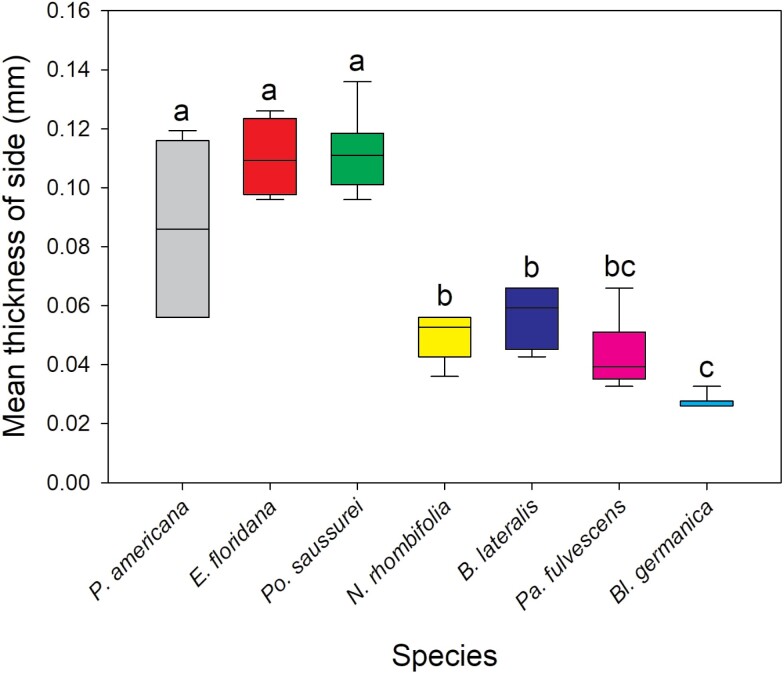
Kruskal–Wallis test and Dunn’s post hoc comparison of mean oothecal cuticle side thickness. Boxes that do not share letters are significantly different with *P* ≤ 0.05. Number of oothecae measured (replicate): *P. americana* = 6, *E. floridana* = 4, *Po. saussurei* = 8, *N. rhombifolia* = 5, *B. lateralis* = 6, *Pa. fulvescens* = 6, and *Bl. germanica* = 6.

**Fig. 4.**
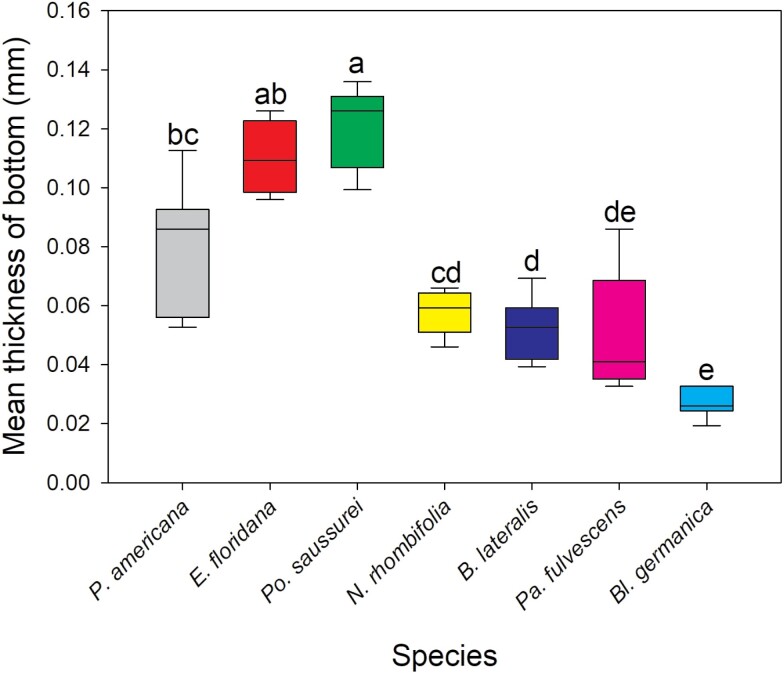
Kruskal–Wallis test and Dunn’s post hoc comparison of mean oothecal cuticle bottom thickness. Boxes that do not share letters are significantly different with *P* ≤ 0.05. Number of oothecae measured (replicate): *P. americana* = 6, *E. floridana* = 4, *Po. saussurei* = 8, *N. rhombifolia* = 5, *B. lateralis* = 6, *Pa. fulvescens* = 6, and *Bl. germanica* = 6.

Cuticle thickness alone is unlikely to be a barrier to parasitism for *A. hagenowii* as the mean ovipositor length (0.92 mm  ± 0.012 mm) was far longer than the thickest cuticles measured. The hardness or flexibility of the cuticle may prevent the ovipositor from puncturing an ootheca, but these aspects of the cuticle have yet to be studied. The oothecal cuticles of the Corydiidae species used in this study felt particularly thick and sturdy compared to the cuticles of Ectobiidae cockroaches, which were thin and easily broken when handled. The cuticles of Blattidae oothecae ranged in thickness and flexibility between the 2 other families (Figs. 2–4).

Several molecules (eg, calcium oxalate and protocatechuic acid) associated with the oothecae and exuviae of *P. americana* act as kairomones for *A. hagenowii* host location and acceptance (Vinson and Piper 1986). Differences among cockroach species in the presence and quantity of these molecules are not well studied, but Kramer et al. (1991) found significantly higher proportions of calcium oxalate in the oothecae of the known hosts *P. americana*, *P. fuliginosa*, and *B. orientalis* (7% to 8%) compared to the nonhost *Bl. germanica* (<1%). The proportion of calcium oxalate in other species’ oothecae has yet to be explored and is an area of particular interest when trying to determine why *A. hagenowii* prefers the oothecae of blattid species over those from other families.

The environment within the ootheca, especially moisture content, may play a more important role than the oothecal cuticle for *A. hagenowii* success. The host species that *A. hagenowii* performed best against (*P. americana* and *B. orientalis*) require little to no absorption of moisture from the surrounding environment to complete development (Roth and Willis 1955). Conversely, oothecae collected from *Pa. lata*, *Pa. fulvescens,* and *N. rhombifolia* quickly shriveled upon removal from their moist substrate, and the oothecae of *Bl. germanica* also desiccated rapidly once removed from the mother. The oothecae of *Po. aegyptiaca*, *Po. saussueri*, and *A. bolliana* contained almost no fluid surrounding the eggs. Roth (1967) noted that the oothecae of *Po. aegypticaca* and 3 species of *Arenivaga* had about half the water content (32% to 37%) of *B. orientalis*, *N. rhombifolia*, and numerous *Periplaneta spp*. (59% to 67%). However, water content alone does not appear to be a deciding factor as the oothecae of *S. longipalpa* (a non-host) maintain a high fluid content throughout their development (Roth 1967).

Information on the differences in the composition of cockroach eggs is severely lacking. For example, studies of *Blattabacterium* spp. cockroach symbionts have found most, if not all, species of cockroach carry a unique strain of the bacterium (Patiño-Navarrete 2013, Noda et al. 2020). Blattabacterium is transmitted from one generation of cockroaches to the next during formation of the egg within the mother. Thus, it is also present during larval development of the parasitoid (Lambiase et al. 1997). *Blattabacterium* spp. do not appear to be pathogenic, but how they or other symbionts may affect the development of *A. hagenowii* larvae as they consume host eggs is unknown. Likewise, the influence that egg nutritional composition may have on *A. hagenowii* development is also not fully understood. Ultimately, determining what factors prevent *A. hagenowii* from utilizing one host species versus another will require gaining a better understanding of the cockroach ootheca beyond the physical barrier of the cuticle.
